# Supplementary material for: Visual working memory-related corrective saccade biases are amplified by task demands, without updating working memory content
Source: Atten Percept Psychophys. 2026 Apr 27;88(5):117. doi: 10.3758/s13414-026-03264-1 (PMC13121585; doi:10.3758/s13414-026-03264-1)
Supplement: Supplementary file 1 — Supplementary file1 (PDF 820 KB) [file 13414_2026_3264_MOESM1_ESM.pdf]

Supplementary Information to:
Visual working memory-related corrective saccade biases are amplified by task demands, without updating working memory content

Patrik Polgári & Alexander C. Schütz
Sensorimotor Learning Unit, Department of Psychology, University of Marburg, Germany

Redefinition of color coordinates:

Hollingworth & Luck's (2009) original paradigm used similar memory and foil color hues in the test phase of the VWM task. When trying to recreate the paradigm as our "Difficult condition" with the colors used in the original study (defined in the 1931 CIE color coordinate system), some color pairs were easier to distinguish than others, making the task's difficulty uneven throughout trials. Thus, we chose to use new sets of color coordinates such that their coordinates in the CIE lab coordinate system are equidistant within a color set (*e.g.*, 5 blue values), making them perceptually equidistant. To achieve an even difficulty between trials, in the memory test phase of the Difficult condition we always used pairs of colors that were neighboring each other within a color set (*e.g.*, "red2" and "red3"). In the Easy condition, a strict control of the choice of exact color values was not necessary, since colors from different categories (*e.g.*, a blue and a red) were presented in the test phase.

List of CIELab coordinates of the colors used as memory targets/foils in the VWM task:

'red1': [50.0, 72.0, 20.0],
'red2': [50.0, 70.0, 22.0],
'red3': [50.0, 67.0, 33.0],

'red4': [50.0, 64.0, 40.0],

'red5': [50.0, 57.0, 46.0],

'blue1': [37.7, 14.2, -74.7],

'blue2': [37.7, 19.2, -72.7],

'blue3': [37.7, 24.2, -70.7],

'blue4': [37.7, 32.2, -68.7],

'blue5': [37.7, 40.2, -64.7],

'green1': [64.3, -72.0, 26.5],

'green2': [64.3, -68.0, 31.5],

'green3': [64.3, -64.0, 39.5],

'green4': [64.3, -58.1, 44.5],

'green5': [64.3, -52.1, 52.5]

List of CIELab coordinates of the colors used in the stimulus array in the Fixation task:

'red': [48.134, 76.002, 68.356],

'blue': [38.551, 59.762, -100.418],

'green': [38.725, -43.373, 45.623],

'yellow': [91.819, -18.197, 97.355],

'magenta': [60.782, 93.143, -66.044],

'black': [0.005, -0.019, 0.008],

'white': [100.000, 0.000, -0.000],

'brown': [38.021, 11.268, 33.511],

'pink': [66.749, 41.524, 8.617],

'orange': [59.798, 45.019, 71.724],

'aqua': [87.969, -41.583, -20.914]

Figure: Main results with individual datapoints.

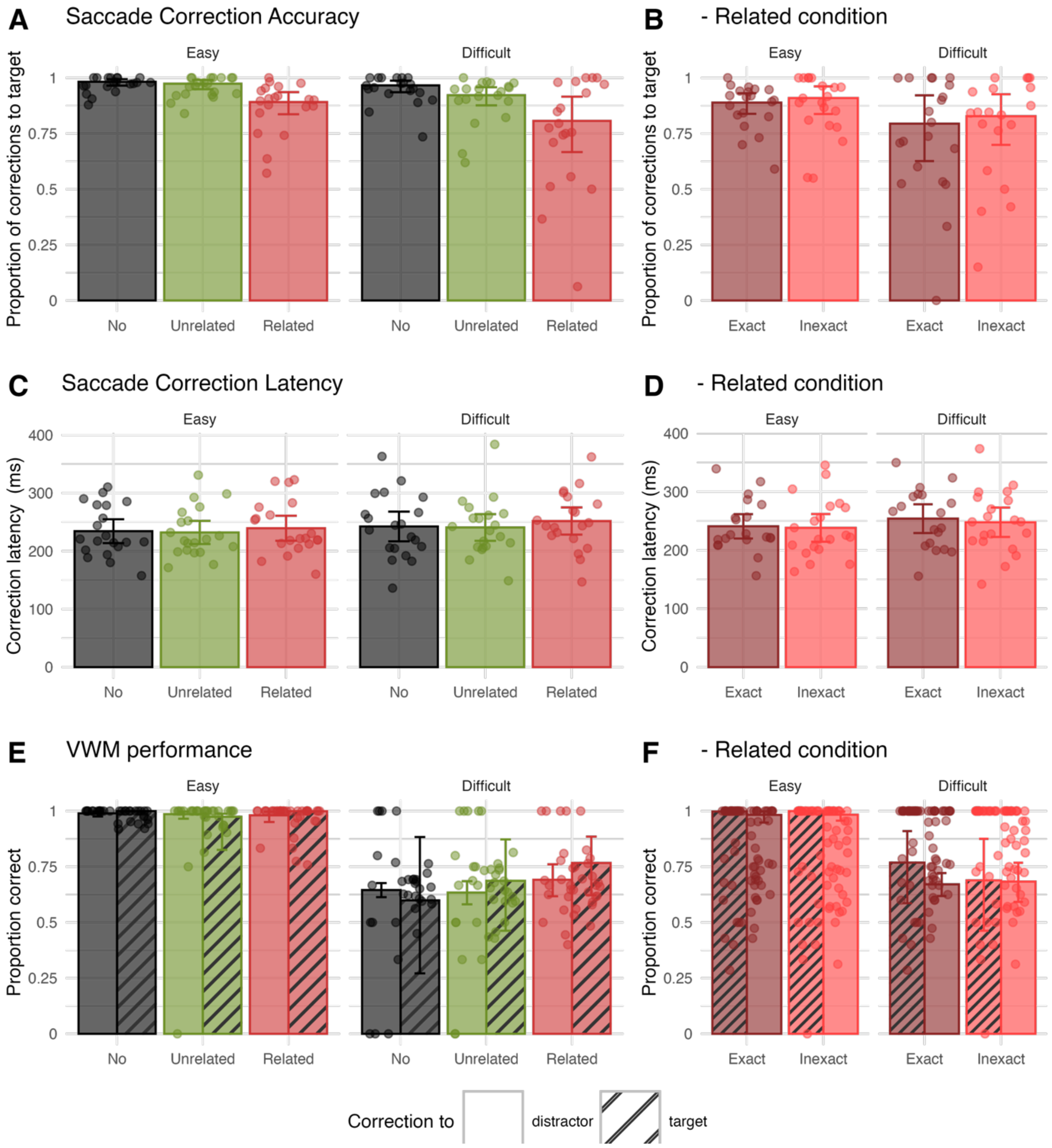

Average saccade correction accuracy, average latency of correct saccade corrections to the saccade target, and average performance in the VWM task by difficulty levels in the three distractor color change conditions (A, C, E) and in the two sub-conditions in the Related change condition only (B, D, F). In the graphs on VWM performance, empty bars correspond to trials where the saccade was corrected towards the saccade target and striped bars correspond to trials with corrections towards the distractor. Note that while statistical analyses on proportion data were conducted on arcsine square root transformed data, the figure represents back-transformed averages ±95%

confidence intervals (error bars), and non-transformed individual data points (circles). Note that the y-axes have a different scales here than in the main manuscript.

Additional model fitting including saccade latency.

In an attempt to further explore the possible mechanisms and understand which factors play a role in the VWM performance in our task, we fitted logistic mixed models which include corrective saccade latency. In fact, in studies investigating saccadic selection in VWM content, shorter saccade latencies were associated with better WM performance (Ohl et al., 2024).

Saccade latency was either standardized (z-scored) globally across all participants or standardized within participant before entered as a continuous predictor, to account for both between-participant variability and trial-by-trial variability within participants. In each case we fitted a logistic mixed model relating the binary response given on each trial (0=incorrect, 1=correct) to the four factors (response ~ difficulty + change condition + correction direction + saccade latency + (1|subject).

Comparing our base model with the one including globally standardized saccade latency indicated a tendential improvement of our base model by the addition of saccadic latency (AIC_{base}=4087.3, $AIC_{latency\_global}$=4086.3, χ^2=3.08, p=0.08). Models including an interaction between saccade latency and the base factors indicated no improvement of model fit (all p-values > 0.05).

Next, comparing the base model with the one including within-participant standardized saccade latency showed no improvement of model fit (AIC_{base}=4087.3, $AIClatency\_within\_part.$=4087.7, χ^2=3.08, p=0.21). These results indicate that the tendential improvement of model fit with the inclusion of saccade latency reflects between-subject variability, but trial-by-trial variability within participants could not be accounted for. In other words, while there seems to be somewhat considerable variability between participants in their performance and corrective saccade latencies, quicker corrective saccades do not seem to be related to better VWM performance in our task.

Additional model fitting in the Difficult condition only.

Given the high accuracy in the Easy condition, we repeated the model fitting separately in the Difficult condition (which contained more balanced numbers of trials where saccade correction occurred toward the target vs. distractor than the Easy condition) to exclude the possibility that an effect is masked by the ceiling effect in the Easy condition.

We compared an additive model condition (response ~ change condition + correction direction + (1|subject) with one that contained an interaction effect between change and corrective saccade direction (response ~ change condition * correction direction + (1|subject). Model comparison showed that the fit was not improved by the addition of interaction term ($AIC_{no\text{-}interaction}$=3362.7, $AIC_{interaction}$=3366.2, χ^2=0.42, p=0.81), favoring the more parsimonious model. The estimates of fixed-effect (and interaction effects in the corresponding model) were all small in magnitude ($|\beta| < 0.18$) and their 95% confidence intervals included zero. These results corroborate those of the ANOVAs in the Difficult condition only, consistent with an absence of meaningful effect of change condition or saccade correction direction on VWM performance.

References

Hollingworth, A., & Luck, S. J. (2009). The role of visual working memory (VWM) in the control of gaze during visual search. *Attention, Perception, & Psychophysics, 71*, 936–949. https://doi.org/10.3758/APP.71.4.936

Ohl, S., Kroell, L. M., & Rolfs, M. (2024). Saccadic selection in visual working memory is robust across the visual field and linked to saccade metrics: Evidence from nine experiments and more than 100,000 trials. *Journal of Experimental Psychology: General, 153*(2), 544–563. https://doi.org/10.1037/xge0001520
